# A unified framework for local-ancestry-aware genetic association analysis across biobanks

**DOI:** 10.64898/2026.08.09.26360047

**Published:** 2026-08-11

**Authors:** Linfeng Hu, Taotao Tan, Kai Yuan, Ying Wang, Bram L. Gorissen, Yi-Sian Lin, Pragati Kore, Wenhan Lu, Ravi Mandla, Zhuozheng Shi, Kangcheng Hou, Konrad J. Karczewski, Hailiang Huang, Benjamin M. Neale, Mark J. Daly, Alicia R. Martin, Bogdan Pasaniuc, Elizabeth G. Atkinson, Wei Zhou

## Abstract

Biobanks increasingly include individuals with admixed genomes, yet conventional genome-wide association study frameworks either exclude participants who cannot be confidently assigned to a discrete ancestry group or ignore ancestry-specific effects. We present FELIX, a scalable framework for local-ancestry-aware genetic analysis that retains all participants without requiring discrete ancestry assignment. FELIX combines a compact ancestry-resolved genotype representation (FELIXla) with an adaptive association test that jointly evaluates shared-effect and ancestry-specific models at each variant (FELIXassoc). Simulations demonstrated well-calibrated inference under case-control imbalance and power that adapted to the locus-optimal model. Across 24 phenotypes in 240,038 All of Us participants, FELIX analyzed the 12.1% of individuals excluded by global-ancestry clustering and identified 15.4% more genome-wide significant loci than global-ancestry meta-analysis. Additional discoveries arose from recovering ancestry-specific haplotypes carried by admixed participants and from detecting ancestry-dependent marginal effects. Full-cohort effect estimates also improved polygenic score prediction across ancestries and traits.

## Full Text Availability

The license terms selected by the author(s) for this preprint version do not permit archiving in PMC. The full text is available from the preprint server.

